# Variation in encoding context benefits item recognition

**DOI:** 10.3758/s13421-024-01603-x

**Published:** 2024-07-02

**Authors:** Jefferson Salan, Devyn E. Smith, Erica S. Shafer, Rachel A. Diana

**Affiliations:** 1 Department of Psychology, Virginia Tech, 890 Drillfield Dr., Blacksburg, VA 24061 USA; 2 Department of Psychology, University of Virginia, Charlottesville, VA USA

**Keywords:** Episodic memory, Recognition, Context effects

## Abstract

The current study assesses whether varying the encoding context of a repeated event is a potential strategy to improve recognition memory across retrieval contexts. Context variability, also known as encoding variability, has historically been investigated primarily using recall and cued recall tasks, with the consensus being that encoding variability is not necessarily beneficial for episodic retrieval. However, recent studies (see text) suggest that test type may determine the strategy’s effectiveness. Aligned with these recent findings, we found consistent benefits to simple item recognition when a word was studied in more variable contexts compared to less variable contexts across four experiments. This main effect of context variability occurred when crossed with a manipulation of repetition spacing and when crossed with a manipulation of encoding-retrieval context match. Variation in encoding contexts beyond the future retrieval context led to better item recognition than repeated study exposures within the future retrieval context. We argue that the current study and other recent findings indicate a need to re-evaluate the historical consensus on encoding variability as a beneficial strategy for learning.

## Introduction

Episodic memory has at least two unique properties among neural memory systems: single-trial learning of information that can be consciously retrieved and the incorporation of event context into its representations. The first property, encoding speed, indicates that episodic memory is likely critical for learning, both in daily life and in the classroom (Greenberg & Verfaellie, 2010). Although episodically encoded information can be incorporated into the semantic memory system over time and repeated experiences, the semantic memory system does not appear to allow single trial learning (Cooper et al., 2019; McClelland et al., 1995; see Coutanche & Thompson-Schill, 2014, for an alternative view). The second property, contextual information, is often irrelevant to the primary goals of learning (e.g., creating semantic knowledge), but may still influence the success of initial learning via the episodic system. The studies reported here investigate whether context can be manipulated to increase recognition memory. These studies are intended to both elucidate properties of the episodic memory system and identify strategies that can support learning.

The context of an event can be identified in several ways, but perhaps the most objective is that context features of events change slowly across time. For example, a series of events, like studying individual muscle names and locations for an anatomy course in a library, would have a number of features in common that are slow to change: location, room dimensions, furniture, mood, clothing, temperature, etc. These features are the context of the events. Each event also has features distinct from the other events that are fast-changing: the names of the muscles, their locations in the body, and any thoughts or emotions generated in response to those features. These fast-changing features are the focus of the events or the “items.” Later recognition of an item (muscle name) does not require retrieval of the contextual information (location, mood, etc.), if a judgment is made using familiarity alone. Nonetheless, previous studies have demonstrated that the context of an event affects the nature of the item information that is encoded and subsequently interacts with retrieval conditions to determine recognition success (e.g., Tulving & Thomson, 1973). Tulving and Thomson’s experiment (1973) asked participants to recognize individual items that had previously been studied as word pairs (e.g., glue-chair). They found substantially decreased memory when the test items were presented in a different context (a different paired word, e.g., table-chair) rather than the original context. Findings of this type indicate that memory for an event or item can be influenced by the associated context information, regardless of whether that context information is relevant to the task being performed.

Tulving and Thomson (1973) defined the principle of “encoding specificity” based on the finding described above: that recognition performance improves when an encoding experience and a retrieval experience are more similar but declines when an encoding experience and a retrieval experience are different from one another. A similar principle has been defined in terms of processing rather than context by Morris and colleagues (1977), which they called “transfer-appropriate processing.” Both principles agree that a highly effective encoding strategy would be to encode an item by processing it in the same way, or experiencing it in the same context, as the eventual retrieval scenario. We will refer to this strategy with the more general term “encoding-retrieval match.” Unfortunately, this strategy assumes that the eventual retrieval scenario is known in advance and that retrieval will be limited to a single scenario. Encoding-retrieval match cannot be implemented when the retrieval context is unknowable in advance. For example, one might consider a college student preparing for an exam. The particular type of question, its wording, and the connections to other concepts are all factors that influence the likelihood of retrieving the correct information on the exam. However, the student is not typically told those details before the exam and cannot study in a way that specifically matches the test context. For that same college student, information being learned in a class that is critical to job function throughout their career needs to be flexibly retrieved in a variety of circumstances, rather than limited to a single retrieval context. Thus, strategically applying encoding-retrieval match may not be beneficial in these real-world circumstances. However, it may be possible to improve the *probability* that features experienced during encoding will be reinstated within an as-yet-unknown retrieval context.

We propose that an unknown, or flexible, retrieval context may be strategically matched by broadening the range of contextual features/cues or processes accessed during encoding. Indeed, it has long been recognized that episodic memory retrieval is driven by cues, either encountered in the environment or effortfully reinstated, that bring to mind events and information experienced previously (Bjork & Bjork, 1992). The more cues encoded and the broader the range of those cues, the more likely some of those cues will match the unknown retrieval circumstances. The idea of increasing variety or breadth during encoding has been termed “elaboration” (Anderson & Reder, 1979), but the current study manipulates elaboration of stimuli by controlling encoding variability/context variability.

### History of encoding variability research

Variability was identified as an important factor in learning early in the scientific study of cognition (Estes & Burke, 1953). The variability of contextual factors during encoding has previously been identified as a potential mechanism for the spacing effect (see Karpicke et al., 2014). The spacing effect is the robust finding that increasing the temporal lag and amount of intervening information between two encoding experiences will increase the likelihood of later retrieval. This is sometimes investigated as the distinction between short spacing (at least one intervening item between study exposures) and long spacing, which produces improved retrieval with increased distance (Delaney et al., 2010).^1^ An encoding variability account of spacing emphasizes the differences in temporal context from an item’s first exposure to the next, implying that an increased range of cues are stored with the item representation when repetitions are spaced apart compared with when they are spaced more closely (Melton, 1970).

The encoding variability explanation for the spacing effect was challenged when a key prediction, that the spacing between unrelated words should predict their aggregate memory performance in the same way as for a repeated item, was initially not supported (Ross & Landauer, 1978). However, recent research by Lohnas and colleagues (2011) has refuted that conclusion, and indicated in multiple datasets that two unrelated words spaced more distantly are indeed better remembered in aggregate than two unrelated words spaced more closely during study (Lohnas et al., 2011). Therefore, encoding variability cannot be ruled out as a mechanism for the spacing effect on the basis of Ross and Landauer’s (1978) conclusions.

Encoding variability was further challenged as an explanation for the spacing effect because studies that simultaneously manipulated encoding variability and spacing found that encoding variability provided a consistent benefit to free recall or cued recall only for items studied via back-to-back repetitions (e.g., Greene & Stillwell, 1995; Verkoeijen et al., 2004). The lack of an encoding variability benefit for spaced items in free-recall and cued-recall paradigms has been interpreted as indicating that variability cannot explain the spacing effect. An alternate interpretation might be that variability can be induced via direct manipulation *or* via spacing, but that two manipulations of variability do not produce double the benefit. An additional consideration is that we are not aware of any prior studies that have manipulated both spacing and encoding variability followed by a simple recognition test, which we address in the current study in Experiment 2. For both of these reasons, we propose that encoding variability should not yet be ruled out as a contributing mechanism for the spacing effect.

### Encoding variability as a manipulable strategy

Regardless of its role in the spacing effect, the current study examines whether encoding variability is beneficial for memory in general. This question arises from the proposal that an increase in the variety of features encoded for an event (and therefore an increase in the possible retrieval cues that will be relevant under unknown or flexible retrieval circumstances) should benefit recognition. If this is true, it may be a broadly applicable strategy across multiple experimental paradigms. A few prior studies have directly manipulated contextual cues or encoding processing across repetitions of a stimulus.

Recent work by Zawadzka and colleagues (2021) extensively tested the outcomes of encoding variability on free-recall and cued-recall performance. They found no benefit for variable processing of items (operationalized via processing questions during study) when memory was tested with a free-recall test, consistent with earlier investigations (Postman & Knecht, 1983) but conflicting with at least one other study (Huff & Bodner, 2014). However, when some form of semantic cueing was used at test, an encoding variability benefit was found. If non-semantic recall cues were provided (i.e., rhyming cues), there was no effect of variability. The authors concluded that varying the processing questions used during encoding led to emphasis of varying semantic features of the stimuli. Therefore, memory benefits only appeared when semantic features were also emphasized during retrieval and not when contextual or inter-item relationship features were emphasized (as may be the case in free recall).

Beginning with Opitz (2010), the idea that manipulations of encoding variability can affect item recognition success, rather than recall or cued recall, has been an increasing topic of interest in the literature. These previous investigations of encoding variability using recognition paradigms have produced relatively consistent results indicating a benefit for encoding variability on recognition success, but have sometimes suffered from confounding factors. For example, Huff and Bodner (2014) found an encoding variability benefit for both free recall and item recognition; however, the item recognition test always followed an initial free recall test on the same items. This raises the possibility that recognition performance was influenced by the recall task.

Other studies that have found an encoding variability benefit for recognition have not controlled repetition spacing (Opitz, 2010; Sievers et al., 2019). Therefore, the benefit of encoding variability in these studies may have been driven by the well-established benefit of distant repetition spacing in the variable encoding conditions as compared to closer repetition spacing in the consistent encoding conditions. In order to examine this issue, Experiment 1 in the current study is essentially a conceptual replication of the Sievers et al. paper, whereas Experiment 2 assesses whether encoding variability interacts with repetition spacing.

The clearest evidence currently available regarding encoding variability across repetitions and its effects on item recognition comes from a recent study by Zhang and Hupbach (2023), which controlled repetition spacing. The authors manipulated conceptual variability by presenting encoded items (object pictures) with either the same encoding question or different encoding questions across study repetitions (e.g., “fits in a shoe box?” and “is a tool?”). At test, participants were asked to make old/new judgments in Experiment 1B and to make old/similar/new judgments, followed by a source memory test, in Experiment 2B.^2^ The results indicated that conceptual variability improved item recognition memory in Experiment 2B but there was no effect of variability in Experiment 1B. The authors identified two potential explanations for the inconsistency in results between these experiments. First, they suggested that requiring source memory judgments in Experiment 2B may have encouraged participants to rely on recollection in their initial item memory judgments, whereas Experiment 1B may have encouraged participants to rely on familiarity. Second, they proposed that their item recognition findings may have been limited by ceiling effects in Experiment 1B when both the consistent and the variable context conditions produced hit rates above 90%. The consideration of ceiling effects is particularly important in studies of repetition and encoding variability because repetition itself increases performance substantially. We address the role of ceiling effects by adding a delay between encoding and retrieval in Experiment 4.

Overall, existing evidence suggests that item recognition may benefit from contextual variability during encoding, but issues with repetition spacing and ceiling effects prevent us from drawing strong conclusions from the existing literature.

### Nature of the variability manipulation

The term *encoding variability* has not always been defined in the same way across the literature. Some definitions of encoding variability refer specifically to the spacing effect and ignore its potential application to other paradigms. For example, encoding variability has been referred to as the idea that “as the lag between repetitions increases, the memorial representations approach independence” (Bray et al., 1976, p. 548). Other researchers apply the term more broadly and use it to encompass variability due to both item-level changes and context-level changes (e.g. Benjamin & Tullis, 2010).

One group of researchers has drawn a distinction between encoding variability and context variability, arguing that context variability benefits item memory and is a partial mechanism for the spacing effect, whereas encoding variability does not benefit item memory (Karpicke et al., 2014). They define encoding variability as “the idea that when items or materials are experienced multiple times, the materials are encoded in different (variable) ways during each encounter, and this is assumed to increase the number of retrieval routes a person has to access material in the future” (Karpicke et al., 2014, pp. 251–252). They propose that this variability is different from contextual variability, which they define as “the specific idea that different temporal/contextual features can be encoded as part of the representation of repeated events” (Karpicke et al., 2014, p. 252).

It is unclear if the differences identified by Karpicke and colleagues between encoding variability and context variability are critical in determining any effect on item memory. In our opinion, it is likely that by changing context features one may also affect the features of the item that are encoded, as proposed by the encoding specificity principle (Tulving & Thomson, 1973), and therefore it may be difficult to define encoding variability and context variability independently. Nonetheless, the current study is focused on variability in contextual features rather than item-specific features and, therefore, we have chosen to refer to the manipulation used in the current study as “context variability.”

### Returning to encoding-retrieval match: Retrieval cues

Prior studies of the effect of context variability on item recognition have not manipulated the corresponding retrieval context. As described above, context variability as a strategy may be most useful when the retrieval context is unknown. The current study explicitly tests this question in Experiments 3 and 4 in order to begin to identify potential mechanisms for any benefit of content variability. If variability does produce a benefit to item recognition, then it might achieve this benefit by increasing the likelihood that cues from the encoding experiences will overlap with the retrieval context. In other words, context variability potentially increases the likelihood of a match between encoding and retrieval contexts. If so, purposely reinstating the encoding context during retrieval (encoding-retrieval match) should make context variability irrelevant to memory performance. However, if context variability further benefits memory even when an encoding-retrieval match occurs, there may be additional mechanisms involved.

Finally, although transfer appropriate processing and encoding specificity are robust, replicable, properties of episodic memory, it is unclear whether the relative proportion of encoded information that matches future retrieval cues affects memory performance. That is, given a large or small set of cues, both of which include cues matching retrieval, a smaller set that is strengthened via repetition may benefit memory (e.g., Dennis & Humphreys, 2001). A strict interpretation of encoding specificity or transfer-appropriate processing seems to suggest that variability beyond implementation of an encoding-retrieval match might be harmful to recognition. We address this question in Experiment 4.

## Experiment 1

This experiment investigates whether manipulating cognitive context variability has benefits for item memory, as tested in a recognition paradigm. It serves as a conceptual replication of the study by Sievers and colleagues (2019).

### Method

#### Participants

Forty-eight members of the Virginia Tech community participated in the experiment in exchange for extra credit in psychology courses. Data files from two participants (one from encoding question set 1 and one from encoding question set 2) were lost due to computer error. Participants were pseudo-randomly assigned to conditions for the between-subjects manipulation of encoding questions such that encoding question sets 1 and 2 had a final N of 11 while encoding sets 3 and 4 had a final N of 12. Participants’ ages ranged from 18 to 23 years, with an average age of 19 years. Thirty-five participants were female. Sample size was chosen arbitrarily rather than via power analysis.

#### Design and materials

**Fig. 1:**
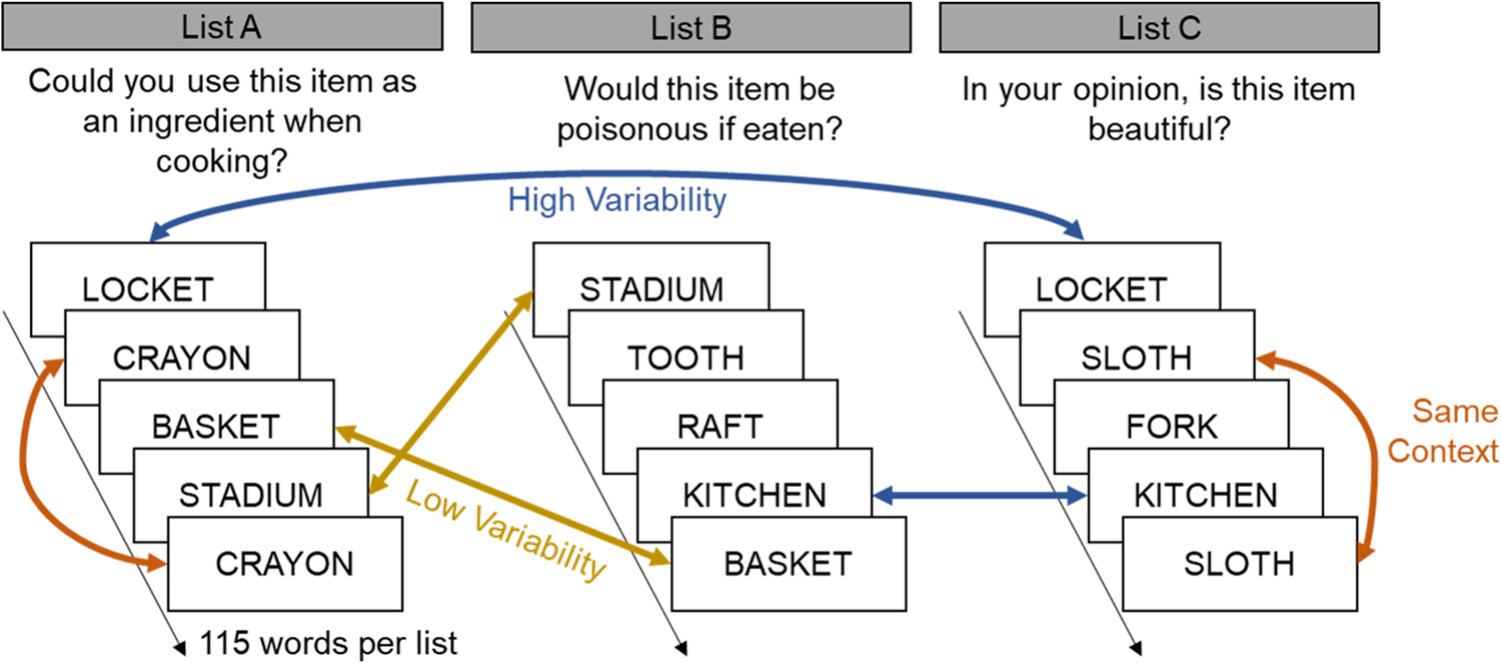
Experiment 1 design and sample trials from encoding question set 3

Experiment 1 used a 4 × 3 mixed design with the factor “encoding question sets” (four levels) manipulated between-subjects and the factor context variability (High, Low, or Same) manipulated within-subjects. High Variability was defined as one study exposure in one of the similar contexts (List A or List B, which used the same or similar encoding questions) and one study exposure in the unique context (List C, which used a distinct encoding question). Low Variability was defined as one study exposure in each of the two similar contexts (Lists A and B). Same Context was defined as two study exposures on the same list (two presentations in List A, B, or C). Figure 1 shows a schematic representation of the design of Experiment 1. For this initial study, repetition spacing was not controlled and was therefore shorter in the Same Context condition than in the High or Low Variability conditions. The study order of the three lists was counterbalanced across subjects.

We chose to define context in episodic memory via its slowly changing nature in comparison to the faster-changing nature of individual items or events. In this experiment, the slowly changing Lists A, B, and C were temporally blocked sets of trials that used a consistent encoding question. In between each list, the participant briefly left the testing room and interacted with the experimenter. Therefore, although the study item changed on each trial, the context changed only twice within the study phase of the experiment (between the first and second lists and between the second and third lists). It should be noted that other definitions of context do not require a more slowly changing signal than the event, and that context variability has previously been studied with randomly assigned contexts that change at the trial level rather than the block level (e.g., Zhang & Hupbach, 2023). The current study cannot determine whether these methods of context manipulation produce different variability effects.

The encoding question sets used to manipulate context variability are shown in Table 1. Question sets 1 and 2 used the same encoding question for the similar lists (A and B) but a different question for the unique list (C). Question sets 3 and 4 used conceptually similar questions, referring to the same property of the target item, for Lists A and B with a distinct question for List C. Thus, the Low Variability conditions were more similar to one another for encoding question sets 1 and 2 than for sets 3 and 4.

**Table 1:** Encoding questions used in between-subjects conditions 1 through 4, specified according to List A, B, or C. Lists A and B served as similar contexts to one another whereas List C served as a unique context

Encoding question set	List A	List B	List C
1	Do you find this item to be pleasant	Do you find this item to be pleasant	Would this item fit in a shoe box?
2	Would this item fit in a shoe box?	Would this item fit in a shoe box?	Do you find this item to be pleasant
3	Could you use this item as an ingredient when cooking?	Would this item be poisonous if eaten?	In your opinion, is this item beautiful?
4	In your opinion, is this item beautiful?	Do you think this item is unpleasant-looking?	Could you use this item as an ingredient when cooking?

The items-to-be-remembered in this study were randomly selected from a set of 792 concrete nouns selected from the MRC database (Coltheart, 1981). The word set had a mean concreteness rating of 585 (ranging from 538 to 670), mean number of letters of 5.64 (ranging from 4 to 9), and mean Kucera-Francis (Kucera & Francis, 1967) frequency of 22.94 (ranging from 1 to 150).

#### Procedure

Participants were told that the experiment would test their memory and were given an overview of the procedures and their rights prior to signing a consent form. Participants were then given written instructions which were reviewed orally by the experimenter. The instructions stated that they would be asked to study concrete nouns, answering a yes/no question for each word. They were told that the items would be studied in three lists, that many words would repeat, and that they should not try to use strategies to memorize the words other than answering the question asked.

The experiment was programmed via Neurobehavioral Systems’ Presentation software. Each word was shown in 24-point font on the computer screen for 1,500 ms followed by a 500-ms fixation prior to the next item. Participants pressed the “J” key to answer the provided question with “yes” and the “K” key to answer the provided question with “no.” The encoding question was previewed for 3,000 ms at the beginning of each of the three lists and then shown on the screen below each word in 16-point font on each trial. After each of the three study lists, participants were required to open the testing room door and ask for further instructions from the experimenter.

The study lists included 60 target words studied with High Variability, defined as one presentation on either List A or List B (using the same or similar encoding questions) and one presentation on List C (using a unique encoding question). An additional 60 target words were studied with Low Variability, defined as one presentation on List A and one presentation on List B. Finally, 35 target words were repeated within the Same Context (both presentations within the same list). An additional 35 filler words (not repeated) were included in order to create equal list lengths (115 trials per list, see Fig. 1).

Immediately following the three study lists, participants were given written test instructions, which were reviewed orally by the experimenter. They were told that they would see words from all three study lists along with new words that had not been studied. Participants were asked to use the “J” key to indicate that the word had previously been studied in the experiment (Old) and the “K” key to indicate that the word had not been previously seen in the experiment (New). If the item was recognized as Old, participants were then asked to indicate one or more study lists on which the item had been viewed (labeled as study list 1, 2, or 3 according to the order of presentation). They were told to press “Don’t Know” if they could not remember the list or lists on which the item had been seen. These source memory judgments were collected for exploratory analyses but are not described or analyzed in this article. It is important to note that the difficulty of the source memory judgment varied across the High Variability, Low Variability, and Same Context conditions in this experiment, and therefore cannot be compared in the same way as item memory.

Once the instructions were provided, all test items were presented in a single list. Participants were provided a maximum of 10 s to respond to each word, ending when an appropriate key was pressed, with an additional 10 s to make source judgments after an old judgment. The next word prompt appeared after a 500 ms fixation screen. The test items included all 190 previously studied items (35 of which were filler items) as well as 190 new items drawn randomly from the same set of words described above. Upon completion of the test list, participants were debriefed as to the study purpose.

### Results and discussion

The primary dependent measure in this study was the hit rate (proportion of old items correctly recognized as old). False alarms did not vary with the manipulation of context variability and therefore differences in d' would be driven exclusively by the hit rate. We did calculate overall false alarms and d' in order to identify any participants whose memory performance was low. The average proportion of false alarms for question sets 1 through 4 was 0.10, 0.11, 0.13, and 0.09, respectively. A one-way between-subjects ANOVA indicated there were no significant differences among the false alarm rates for the question sets, *F*(3,42) = 0.72, *p* = 0.55, ${\upeta }^{2}$= 0.05. Overall accuracy was high across all participants in Experiment 1, with mean d' (standard deviation in parentheses) for question sets 1 through 4 of 2.71 (0.36), 2.39 (0.48), 2.26 (0.56), and 2.50 (0.38), respectively. All participants’ d' scores were within three standard deviations of the mean for their question set.

**Fig. 2:**
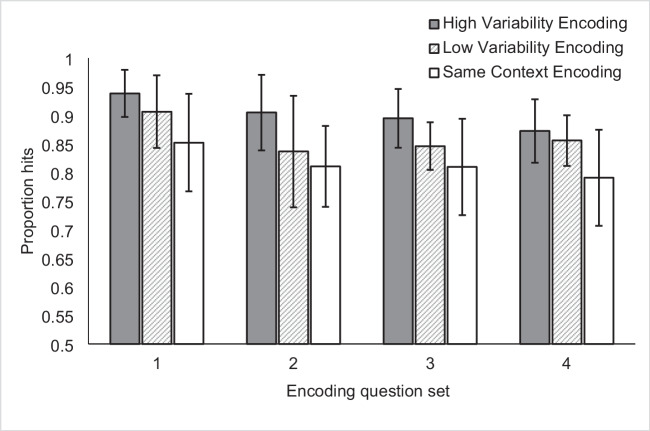
Mean proportion hits in Experiment 1 for the within-subjects manipulation of context variability, as demonstrated in each of the between-subjects encoding question set conditions. Error bars indicate Cousineau-Morey within-subjects 95% confidence intervals for differences due to context variability (Cousineau, 2005; Morey, 2008)

Figure 2 shows the proportion of hits for each context variability condition within each encoding question set. A 3 × 4 repeated-measures ANOVA (three-level within-subjects factor of context variability and four-level between-subjects factor of encoding question set), revealed a main effect of context variability, *F*(2, 84) = 31.59, *p* < 0.001, ${\upeta }_{\text{p}}^{2}$ = 0.43, but no main effect of encoding question set, *F*(3, 42) = 1.68, *p* = 0.19, ${\upeta }_{\text{p}}^{2}$ = 0.11, and no interaction between context variability and encoding question set, *F*(6, 84) = 0.58, *p* = 0.75, ${\upeta }_{\text{p}}^{2}$ = 0.04. We note that sample size for this experiment was chosen arbitrarily rather than via a priori power analysis, and that the study may have been underpowered. Therefore, we will not interpret any null effects from Experiment 1.

Follow-up paired-samples *t*-tests revealed that the main effect of context variability was driven by significant differences between all three conditions (effect sizes are reported as Cohen’s d): High Variability versus Low Variability *t*(45) = 4.39, *p* < 0.001, d = 0.65, High Variability versus Same Context *t*(45) = 8.70, *p* < 0.001, d = 1.28, Low Variability versus Same Context *t*(45) = 3.60, *p* = 0.001, d = 0.53. We conclude from these results that experimentally induced variability in encoding contexts improves recognition memory for items; however, differences in repetition spacing may have contributed to this finding. The effect of context variability was robust to the particular questions used as markers of context.

Importantly, Experiment 1 did not address the issue of repetition spacing. Although spacing for High Variability and Low Variability items was equated at the block level across participants (due to list order counterbalancing), repetitions were spaced more closely in the Same Context condition than in either the High or the Low Variability conditions. There is some evidence that spacing alone cannot explain the findings in Experiment 1 because the effect of context variability was significant for the difference between High Variability and Low Variability, when spacing was equivalent. However, given the robust nature of the spacing effect and the proposal that context variability may be a partial mechanism by which the spacing effect occurs (see Lohnas et al., 2011), we chose to manipulate context variability and repetition spacing independently in our next study in order to assess the relative contributions of these factors.

Although previous studies have discussed these two effects’ relationship to one another, we are not aware of any prior studies that have manipulated both factors in a recognition paradigm. The most similar study used a two-alternative forced-choice frequency judgment paradigm (Greene & Stillwell, 1995), which found a benefit of item-specific encoding variability (changing paired words) for items repeated back-to-back (massed practice) but not for items that were repeated after four to seven intervening items. Studies using recall tests have found mixed results, with multiple studies finding that context variability benefits memory when repetitions are massed but has no effect when repetitions are sufficiently spaced (Smith & Handy, 2016; Verkoeijen et al., 2004). Our repetition spacing manipulation in Experiment 2 did not include massed trials because prior findings are consistent in indicating any context variability benefit for massed repetitions.

## Experiment 2

### Method

#### Participants

Forty-eight members of the Virginia Tech community, 35 female, participated in Experiment 2 in exchange for extra credit in psychology courses. The mean participant age was 19.89 years, ranging from 18 to 25 years. All participants’ performance fell within 3 standard deviations of the mean d' score and therefore none were excluded. An a priori power analysis based on Experiment 1 indicated that a sample size of 30 participants would provide 96% power to detect a similar context variability effect. However, we elected to increase the sample size because we expected that controlling repetition spacing would decrease the size of the context variability effect found in Experiment 1.

#### Design and materials

Experiment 2 used a 3 × 5 within-subjects design with the factors context variability (High Variability, Low Variability, and Same Context) and repetition spacing (0, 1, 2, 3, or 4 blocks of 12 trials intervening between each item repetition). It should be noted that 0 block spacing is not equivalent to massed practice because item order was randomized within each 12-trial block. Therefore, the likelihood that a word would appear on two successive trials was low.

**Fig. 3:**
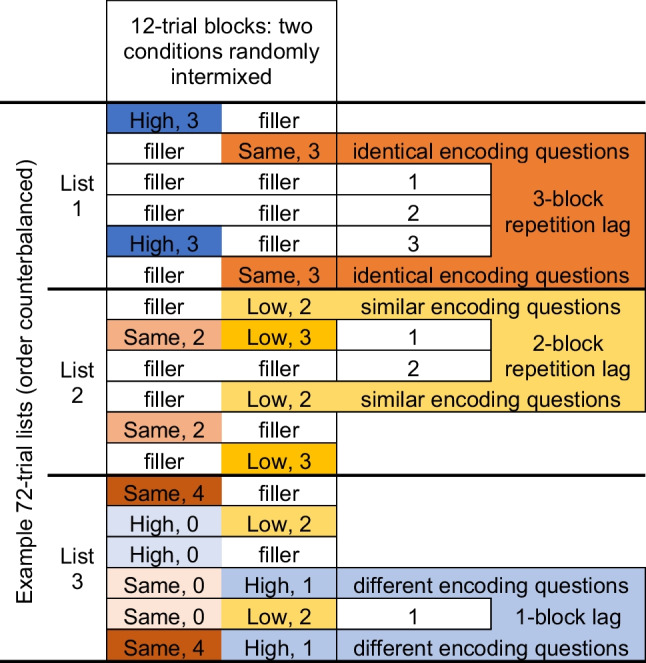
Design schematic for Experiment 2

The study lists were created via custom Matlab scripts using the design shown in Fig. 3. We counterbalanced condition the order of the six study lists (72 trials per list) across subjects. Each list was composed of six blocks of 12 trials. The 12-trial blocks included six words from two different conditions, all of which were studied with the same encoding question during that block. Words were presented in randomized order within a block. Words in the 0-block spacing condition were studied within back-to-back sets of 12 trials. This block-based spacing manipulation combined with randomly ordered items within each block allowed for words in the 0-block spacing condition to occasionally be studied on neighboring trials or with as many as 22 trials intervening. The average number of trials intervening between items in the 0-block spacing condition was 11. Spacing with 11 intervening trials is often considered moderate or long spacing in experiments that investigate the spacing effect.

Due to the complexity of independently controlling spacing and temporally blocked context variability, the four-block spacing condition had fewer trials (six per variability condition) than the other spacing conditions (12 per variability condition). Due to a programming error, participants studied six items in the Same Context 0-block spacing condition and 18 items in the Same Context 1-block spacing condition. All results below are reported as proportions of the total number of words studied in that condition.

Context variability was manipulated using four encoding questions that formed two sets of similar questions. One set referred to the edibility of the object being studied: “Would this item be poisonous if eaten?” and “Could you use this item as a cooking ingredient?” The other set referred to the size of the object being studied: “Could you carry this item in your backpack?” and “Would this item fit in a shoebox?” Items in the High Variability condition were studied once with a question from the edibility set and once with a question from the size set. Thus, participants assessed High Variability items along two different dimensions. Items in the Low Variability condition were studied once with each of the two questions from the edibility set or once with each of the two questions from the size set. Thus, participants assessed Low Variability items along a single dimension but from two different perspectives. Items in the Same Context condition were studied twice with an identical question. The specific questions assigned to conditions were randomized across participants.

Each of 162 target words was studied twice. Those 324 study trials were accompanied by 108 filler words used to induce the required spacing between the target item repetitions. Filler words were studied only once and were not tested. The test list consisted of 162 target words and 162 words that were not viewed in the experiment. All words were randomly selected for each participant from the list of 792 words used in Experiment 1.

#### Procedure

Experiment 2 used the same procedures described for Experiment 1 with the following exceptions. There were six distinct study lists. The encoding question was previewed at the beginning of each 12-trial block rather than at the beginning of each list. After each old judgment, participants were asked to identify the study question or questions that had been presented with the word. The four possible questions were presented along with a “Don’t Know” option as described in Experiment 1. As in Experiment 1, these source memory judgments were collected for exploratory analyses but are not described or analyzed in this article because the difficulty of the source memory judgment varied across the High Variability, Low Variability, and Same Context conditions.

### Results and discussion

Although the primary dependent measure in this study was the proportion of hit responses, we calculated overall false alarms and d' in order to identify any participants whose memory performance was low. The mean proportion of false alarms was 0.08 (SD = 0.07), the mean proportion of hits was 0.82 (SD = 0.11), and the mean d' score was 2.55 (SD = 0.65).

**Fig. 4:**
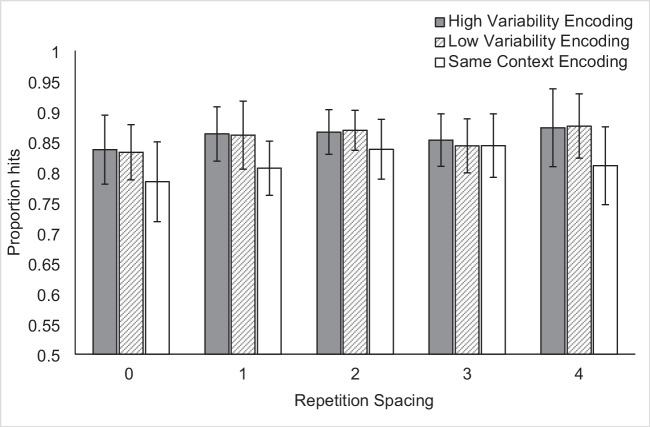
Mean proportion hits in Experiment 2 according to context variability and spacing. Spacing labels indicate the number of 12-item blocks intervening between first and second study presentations of an item. Error bars indicate Cousineau-Morey within-subjects 95% confidence intervals (Cousineau, 2005; Morey, 2008)

Figure 4 shows the proportion of hits for each context variability condition within each spacing condition. A 3 × 5 repeated-measures ANOVA revealed a main effect of context variability, *F*(2, 94) = 10.83, *p* < 0.001, ${\upeta }_{\text{p}}^{2}$ = 0.19, a trend toward a main effect of repetition spacing, *F*(4, 188) = 2.19, *p* = 0.07, ${\upeta }_{\text{p}}^{2}$ = 0.05, and no interaction between context variability and repetition spacing, *F*(8, 376) = 0.57, *p* = 0.80, ${\upeta }_{\text{p}}^{2}$ = 0.01.

Follow-up paired-samples *t*-tests (with effect sizes reported as Cohen’s d) revealed that the main effect of context variability was driven by a significantly lower proportion of hits in the Same Context condition (*M* = 0.82) than in either the High Variability or Low Variability conditions, *t*(47) = 4.06, *p* < 0.001, d = 0.59 and *t*(47) = 3.51, *p* < 0.001, d = 0.51, respectively. There was no difference between the proportion of hits in the High Variability (*M* = 0.86) and Low Variability (*M* = 0.86) conditions, *t*(47) = 0.25, *p* = 0.81, d = 0.04.

**Fig. 5:**
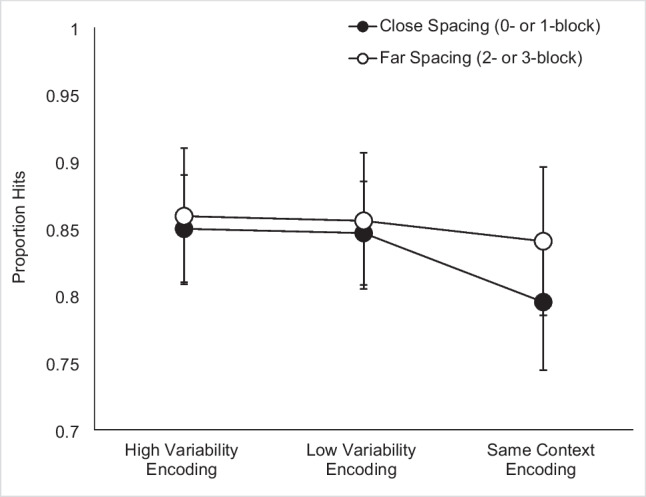
Exploratory visualization of the spacing data in Experiment 2, excluding the four-block spacing condition and viewing the remaining spacing conditions as “close” vs. “far. Error bars indicate Cousineau-Morey within-subjects 95% confidence intervals (Cousineau, 2005; Morey, 2008)

Given the robust nature of repetition spacing effects in episodic memory (Cepeda et al., 2006), we were surprised that the main effect of spacing was not significant. In examining Fig. 4, there appears to be a linear increase in accuracy with spacing for the Same Context condition, with the exception of the four-block spacing condition. Given the comparatively small number of trials in the four-block spacing condition (six per context variability condition) relative to the other spacing conditions (12 per context variability condition), we examined the hit rate standard deviations across all conditions. As speculated, the four-block spacing condition demonstrated the highest standard deviation compared to the other spacing conditions within each variability condition. We created an exploratory visualization of the data in which we dropped the four-block spacing condition and divided the remaining four spacing conditions (0-, 1-, 2-, and 3-block spacing) into two levels: close spacing (0- and 1-block spacing) and far spacing (2- and 3-block spacing). The resulting pattern is shown in Fig. 5 and suggests that the Same Context, close spacing condition has a lower hit rate than any other condition. We interpret this as indicating that variability might be achieved by manipulating the encoding questions (whether via a minimal change in perspective, in the Low Variability condition, or a larger change in the dimension of analysis, in the High Variability condition) or by long temporal spacing (2- or 3-block spacing). However, combining these two factors did not produce a performance benefit beyond the effect of one or the other alone.

This exploratory visualization is consistent with prior claims that context variability is a mechanism that contributes to the spacing effect (e.g., Delaney et al., 2010; Lohnas et al., 2011). However, for the purposes of the current study, the most important finding is that manipulated context variability improves item recognition memory even when spacing is carefully controlled. Experiment 3 tested our primary hypothesis regarding the mechanism by which context variability might benefit item memory: increasing the set of encoded cues and therefore increasing the likelihood of encoding-retrieval match.

## Experiment 3

If encoding-retrieval match is a more important factor in memory than context variability, then matching encoding processing during all study exposures to the upcoming retrieval processing should produce better memory than matching only one study exposure’s processing. Perhaps a student who is told the exact nature of the test questions they will receive should study by practicing that question format repeatedly without any variation. An alternative possibility is that variability has benefits beyond encoding-retrieval match, such that at least one exposure to the test processing (achieving a match) combined with additional variability in processing is more beneficial than repeated practice of the matching retrieval processing. That is, perhaps the student should study the exact nature of the test question at least once, but also study the information in a variety of other ways. Experiment 3 examines the effects of context variability on retrieval success, in both the presence and the absence of encoding-retrieval context match.

### Method

#### Participants

Data were collected from 70 Virginia Tech students, recruited through the Department of Psychology’s research participant pool and from the broader Virginia Tech community. Of the 70 participants, five were excluded from all analyses, for a final sample size of N = 65. One was excluded due to a computer error that did not allow them to complete the test phase, while four others were excluded due to d′ scores below zero in at least one cell of the design. Participants received either extra credit in one of their Psychology courses or $20 as compensation for their time. A minimum sample size of 30 participants was determined by an a priori power analysis (based on the main effect of context found in Experiment 2, 30 participants provides 96% power to detect an effect of the same size). We elected to double that sample size, for a target of 60 participants, given that an interaction between context variability and encoding-retrieval match might be smaller than the main effect of context variability.

#### Design and materials

The experiment used a 3 × 2 factorial design with level of encoding context variability (High Variability, Low Variability, and Same Context)) and retrieval context (Match to the encoding context and Non-match to the encoding context) as the variables of interest. Participants were assigned to one of six counterbalancing schemes to account for differences in condition order. In addition to controlling repetition spacing across conditions, Experiment 3 controlled for cue specificity/cue overload (Watkins & Watkins, 1975). We controlled cue specificity by presenting every encoding question with an equal number of target words on an equal number of trials. Study and test lists were created via custom Matlab scripts.

Word lists for each participant were composed of 360 nouns, four to nine letters each, randomly drawn from a pool of 434 nouns. The word pool was obtained from the SUBTLEXus corpus (normative measures based on film subtitles in American English) and constrained to a SUBTLEXus frequency rating range of 0.02–292.06 words per million (*M* = 6.81) (Brysbaert & New, 2009). Words in the pool were selected for high concreteness ratings, with a range of 4.59–5 (*M* = 4.86, based on a scale of 1 = abstract/language-based to 5 = concrete/experience-based) (Brysbaert et al., 2014). Words also had a minimum prevalence value of 2.00 (a *z*-scored measure of the proportion of people who profess to “know” a word) based on word prevalence norms from Brysbaert et al. (2019).

Of the 360 selected words, half were used as study words and the other half as lure words (randomly selected for each participant). Study words were randomly assigned to either the matching (90 words total) or the non-matching retrieval context condition (90 words). These sets were then further subdivided into thirds, with 30 words randomly assigned to the High Variability, Low Variability, and Same Context conditions for each participant. The study phase included 180 study words, presented three times each, for a total of 540 study trials. The test phase included 360 words, with each of the study and lure words presented once in a random order.

**Fig. 6:**
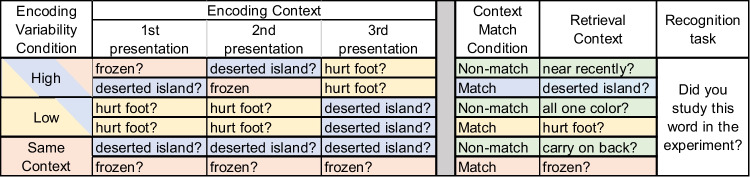
Design schematic for Experiments 3 and 4

A schematic of the experiment design is shown in Fig. 6. Each of the six semantic encoding questions used to manipulate context variability required the participant to think of the item in a specific physical or relational context (i.e., Is this item all one color?; If you were stranded on a deserted island, would this item be useful?; Can this item be frozen in a freezer?; Could you carry this item on your back?; Have you been near this item recently?; Would it hurt if this item fell on your foot?). Three questions were randomly chosen for each participant to appear during encoding. High Variability items were presented once with each of these three encoding questions. Low Variability items were presented twice with one of the three encoding questions and once with a different encoding question. Same Context items were presented three times with the same encoding question.

All conditions averaged 15 intervening items between each encoding repetition, comparable to the 0-block spacing condition in Experiment 2 but with three study exposures per item rather than two. This spacing control induced differences in delay between the last study exposure and the beginning of the test trials across conditions. These differences in delay were controlled by counterbalancing condition order across participants.

During the test trials, all words were first shown with a yes/no semantic question and then participants were asked whether they recognized the word from the study phase (old/new judgment). The yes/no semantic question for items in the Match retrieval context condition was always one of the questions that had previously been asked about that item during encoding. In the High Variability/Match condition, the yes/no question used at retrieval was randomly selected from the three encoding contexts. In the Same Context/Match condition, items were tested with the encoding context that was viewed during all three study exposures. Selection of the yes/no question shown at retrieval for the Low Variability/Match condition involved two additional factors because all Low Variability items were studied with one repeated question and one non-repeated question (for three study exposures). Half of the participants, according to their counterbalancing scheme, initially saw Low Variability items twice with question “A” and then a third time with question “B” (Low Variability/Match, A-A-B order). The other half of the participants initially saw Low Variability items once with question “A” and then the second and third times with question “B” (Low Variability/Match, A-B-B order). For all participants, half of the Low Variability/Match items were tested with reinstatement of the twice-studied context and half with reinstatement of the once-studied context. That is, half of all Low Variability/Match items for each participant were tested with question “A” and half with question “B.” We note that the design of the Low Variability/Match condition retrieval contexts was chosen to balance the relevance of all three encoding exposures within and across participants rather than to test differences in repetition number and order (due to limited trial numbers).

Items in the Non-match retrieval context condition were presented with one of the three remaining context questions (not seen during the encoding phase of the experiment). Both lure and study items were tested equally often with the three questions viewed during encoding or the three novel questions. In contrast to Experiments 1 and 2, participants were not asked to retrieve information about the encoding context during the test.

#### Procedure

At the beginning of the experiment, participants were briefed on the general nature of the experiment and given the instructions for the study phase. They were informed that their task was to study concrete nouns by responding either “yes” or “no” to a question appearing with each word. Participants were informed that some words would be repeated. They were asked not to attempt to memorize the words with any specific strategy, but to simply answer the questions that appeared on screen.

The study phase was divided into sections according to the encoding question being answered for all items within that section. At the beginning of each section, participants saw an encoding question preview for 5 s. Each subsequent trial began with a 500-ms fixation cross, followed by a word in the center of the black screen for 1,500 ms, with the encoding question shown directly above the word. The response options (“J = Yes” and “K = No”) appeared directly below the word. At the end of each block, a message on-screen instructed participants to notify the experimenter that they were finished with the study list.

The test instructions specified that participants would see a mix of studied words and unstudied words along with some familiar yes/no questions and some novel yes/no questions. For each word, they were asked to first answer “yes” or “no” to the question on the screen and then to indicate whether they had previously seen the word in the experiment. Immediately after answering the encoding question, a new screen appeared displaying the same test word with the question “Did you study this word before?” above it and the response options (“Yes” or “No”) below it. Participants had a maximum of 10 s to respond to each of the yes/no and recognition questions before continuing to the next trial.

### Results and discussion

As in Experiments 1 and 2, the primary dependent measure was the proportion of hits. We calculated overall false alarms and d′ scores in order to assess memory performance for each participant. Participants who had a d′ score below zero in any condition were excluded from all subsequent analyses. The mean overall proportion of hits in the final dataset was 0.88 and the mean overall proportion of false alarms was 0.14. The mean d′ was 3.05.

A repeated-measures analysis of variance (ANOVA) was conducted on the proportion of hits. We hypothesized that context variability and encoding-retrieval match would each benefit item recognition. We also predicted that context variability would benefit performance above and beyond the benefits conveyed by encoding-retrieval match. Thus, we expected a significant interaction effect between context variability and context-match. To specifically test for this third hypothesis, even in the absence of a significant interaction, planned comparisons were conducted via paired-samples *t*-tests to examine mean differences between the Low Variability/Match and High Variability/Match conditions, as well as the Low Variability/Non-match and High Variability/Non-match conditions.

**Fig. 7:**
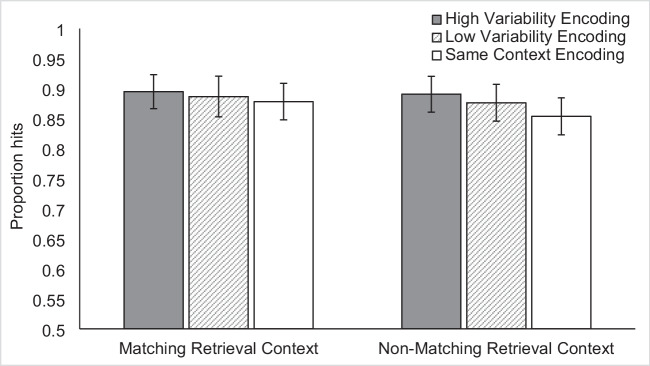
Mean proportion hits in Experiment 3 according to context variability and encoding-retrieval context match. Error bars indicate Cousineau-Morey within-subjects 95% confidence intervals (Cousineau, 2005; Morey, 2008)

The ANOVA revealed the predicted significant main effects of context variability, *F*(2, 128) = 7.00, p = 0.001, ${\upeta }_{\text{p}}^{2}$ = 0.10, and context match F(1, 64) = 4.75, p = 0.03, ${\upeta }_{\text{p}}^{2}$ = 0.07. The overall pattern of hit rates is seen in Table 2 and Fig. 7. The ANOVA did not reveal a significant interaction effect F(2, 128) = 1.10, p = 0.34, ${\upeta }_{\text{p}}^{2}$ = 0.02, suggesting that the effects of both context variability and transfer-appropriate processing on item recognition are independent of one another, contrary to our predictions. Results from the planned paired-samples *t*-tests revealed no significant difference between the Low Variability/Match (*M* = 87.8%) and High Variability/Match (*M* = 89.5%) conditions, *t*(64) = -1.851, p = 0.07, d = 0.23, but they did reveal a significant difference between the Low Variability/Non-match (*M* = 85.4%) and High Variability/Non-match (*M* = 89.1%) conditions, t(64) = -3.625, p < 0.001, d = 0.45. Therefore, the results with respect to the interaction between context variability and encoding-retrieval match are unclear. As demonstrated by the planned comparisons, there was no statistical evidence that variability benefitted memory in the matching context conditions; yet, there was a main effect of variability that was not modified by an interaction with encoding-retrieval match.

**Table 2:** Experiments 3 and 4, mean proportion hits per condition (standard deviation)

Context Variability	Experiment 3		Experiment 4	
	Encoding-Retrieval Context		Encoding-Retrieval Context	
	Match	Non-match	Match	Non-match
High	0.895 (0.104)	0.891 (0.110)	0.904 (0.082)	0.856 (0.104)
Low	0.887 (0.125)	0.876 (0.113)	0.879 (0.103)	0.815 (0.118)
Same Context	0.878 (0.112)	0.854 (0.113)	0.844 (0.111)	0.750 (0.131)

In spite of the lack of immediate clarity that can be gleaned from these results, there is some room for more conclusive interpretation when considering the expectations put forth by a strict interpretation of encoding specificity or transfer-appropriate processing. If encoding-retrieval match is the key factor in recognition success, then encoding an item multiple times within a context that matches the retrieval context (as in the Low Variability/Match condition) should allow for better memory performance than encoding an item in multiple different contexts with only one of those contexts matching the retrieval context (as in the High Variability/Match condition). Yet, our results show no significant difference between the Low Variability/Match and High Variability/Match conditions.

## Experiment 4

Ceiling effects may have had an influence on our findings in Experiment 3. Therefore, we ran a new study to replicate Experiment 3 with a delay of 24 h between the study and test phases of the experiment. We also added a manipulation check during the encoding phase in order to assess the degree to which participants were cooperating with the instructions and therefore being exposed to the context variability manipulation. We preregistered this replication study on the Open Science Framework prior to beginning data collection (10.17605/OSF.IO/SU4DB).

### Method

#### Participants

The target sample size was set based on a GPower 3.1.92 analysis of the effect size for the non-significant interaction term from the Experiment 3 repeated-measures ANOVA. In order to achieve 80% power to detect an effect of the same size, a minimum sample size of 76 was required. Due to the six counterbalancing schemes for condition presentation order, we increased the target sample size to 78 (a multiple of 6).

Participants were recruited through online newsletter advertisements to the Virginia Tech community. We did not exclude participants based on English-language acquisition age or experience, relying on the manipulation check to identify participants who were unable to read the encoding questions and words quickly enough to make accurate responses. Participants received monetary compensation for their time. Data were collected from a total of 101 participants. The average age of the participants was 26 years, with a maximum age of 42 years and a minimum age of 19 years. Self-reported participant gender was distributed as follows: 50 men, 51 women, and 0 non-binary participants. Self-reported participant race/ethnicity was distributed as follows: 3% African, 20% Asian, 1% Black, 5% Caucasian, 1% European, 1% Eastern European, 2% Latinx, 2% Hispanic, 5% Middle Eastern, 21% South Asian, 1% North African, 23% White, and 15% selecting multiple descriptors.

As reported in the project’s preregistration, we set criteria for excluding participants using the manipulation check items during encoding (and overall performance), making exclusion decisions prior to calculating participant means in the conditions of interest. Exclusion criteria are described below after the manipulation check design is explained. Of the 101 participants, 20 were excluded for failing the manipulation check and three were excluded due to performance on the memory test (d' more than 3 standard deviations below the mean, 0.56 or below), for a final sample size of N = 78.

#### Design and materials

The design and materials were identical to those of Experiment 3 with two exceptions. First, a delay of approximately 24 h, with some variability due to scheduling, was instituted between the encoding trials and the retrieval trials. The average delay was 23.81 h, with a minimum delay of 21.67 h and a maximum delay of 26.85 h.

Second, we added trials to each encoding block that served as a manipulation check. The key manipulation in the experiment is the nature of the encoding question presented on each trial; however, the questions are answered based on personal opinions rather than having a clear correct or incorrect answer. Therefore, we have not previously examined responses to the encoding questions. In Experiment 4, in an effort to ensure that participants were attending to the encoding question and answering it in relation to the item being presented, we selected two words for each encoding question that we judged as having a clear correct answer to that question. One word was chosen because it should lead to a “no” answer and the other because it should lead to a “yes” answer for each encoding question. The questions and manipulation check words were: “Is this item all one color?”: storybook (no) and ketchup (yes); “If you were stranded on a deserted island, would this item be useful?”: quicksand (no) and campfire (yes); “Can this item be frozen in a freezer?”: sofa (no) and juice (yes); “Could you carry this item on your back?”: volcano (no) and backpack (yes); “Have you been near this item recently?”: baboon (no) and sidewalk (yes); “Would it hurt if this item fell on your foot?”: yarn (no) and yacht (yes). None of the manipulation check items were shown during the memory test.

Both manipulation check items (“no” and “yes”) were shown once in each encoding block for their relevant question. Therefore, depending on the three questions that were randomly selected to appear during encoding for each participant, some manipulation check items were not shown at all while others were shown either twice or three times. Prior to reviewing any data from the conditions of interest, we examined responses to the manipulation check trials during encoding. We excluded from further analysis any participants whose encoding responses on the manipulation check trials met any of the following criteria: two or more skipped items (out of 14 total manipulation check items), two or more responses that were inconsistent across repetition of the same question/item pair (i.e., answering “yes” to the question/item pair on one trial and “no” to the same question/item pair on another trial), and seven or more responses that we deemed incorrect (regardless of consistency across blocks). Of the 20 participants excluded due to the manipulation check, 11 were excluded due to skipping responses, six were excluded due to inconsistent responses across blocks, and three were excluded due to incorrect responses.

#### Procedure

Other than the addition of 14 un-tested manipulation check items during the encoding phase and a 24-h delay between the encoding phase and retrieval phase, the procedure was identical to that described in Experiment 3.

### Results and discussion

**Fig. 8:**
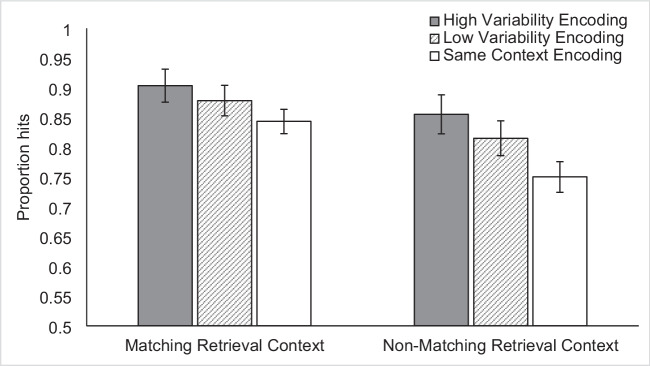
Mean proportion hits in Experiment 4 according to the context variability and encoding-retrieval context match. Error bars indicate Cousineau-Morey within-subjects 95% confidence intervals (Cousineau, 2005; Morey, 2008)

Once again, hit rate was the primary dependent measure for our conditions of interest. We calculated a single d' score, across all trials, in order to exclude participants with low accuracy. The average d' of the 78 participants was 2.05, with a standard deviation of 0.50. This was substantially lower than the average d' in Experiment 3, which was 3.05, as expected given the 24-h delay. The mean proportion of hit responses across all trials was 0.84 (SD = 0.09) and the mean proportion of false alarm responses was 0.18 (SD = 0.12). We concluded that the likelihood of compression in the hit rates due to approaching ceiling was lower in Experiment 4 than in Experiment 3 because the difference in the average hit proportions between the conditions with the highest and lowest values was 0.15 in Experiment 4 as compared to 0.04 in Experiment 3. The means for each condition are shown in Table 2 and Fig. 8.

We performed a 3 × 2 (context variability × context match) repeated-measures ANOVA on the proportion of hits. We replicated the significant main effects of context variability, F(2,154) = 42.22, *p* < 0.001, ${\upeta }_{\text{p}}^{2}$ = 0.35, and context match, F(2, 77) = 137.99, *p* < 0.001, ${\upeta }_{\text{p}}^{2}$ = 0.64, that we found in Experiment 3. The effect sizes were substantially larger than those found in Experiment 3 (variability ${\upeta }_{\text{p}}^{2}$ = 0.10, context match: ${\upeta }_{\text{p}}^{2}$ = 0.07), which we attribute to the removal of participants who did not perform the encoding task as instructed (via the new manipulation check added in Experiment 4) and to the 24-h delay. As noted above, the added delay reduced performance overall and increased the range of performance across the highest- and lowest-performing conditions.

Unlike Experiment 3, we found a significant interaction effect between context variability and context match, F(2, 154) = 5.49, *p* = 0.005, ${\upeta }_{\text{p}}^{2}$ = 0.07. The effect size reveals that this is a small effect in comparison to the two main effects. Follow-up paired comparisons indicated that all encoding-retrieval match differences were significant when compared within the same level of context variability (see Table 2 for condition means and standard deviations). We next compared the Cohen’s d effect sizes for each t-test in order to interpret the interaction. The largest effect size occurred for the comparison between Same Context items tested in Match contexts compared to those tested in Non-match contexts, *t*(77) = 9.10, *p* < 0.001, d = 1.03. The effect sizes of differences due to encoding-retrieval match for Low Variability items (*t*(77) = 6.03, *p* < 0.001, d = 0.68) and High Variability items (*t*(77) = 5.52, *p* < 0.001, d = 0.63) were substantially smaller. Therefore, the benefit of encoding-retrieval match is greater when there is less variability across encoding episodes.

It should be noted that a larger benefit for encoding-retrieval match in the Low Variability condition does not indicate that low encoding variability is preferable when the encoding and retrieval contexts match. Instead, Experiment 4 replicated a key finding from Experiment 3, that multiple repetitions of the encoding context that would later be matched during retrieval (Low Variability/Match *M* = 0.84) produced poorer performance than a single repetition of the encoding context that would later be matched during retrieval in combination with additional non-matching encoding contexts (High Variability/Match, *M* = 0.90, *t*(77) = 5.62, *p* < 0.001, d = 0.64). This indicates that a strict interpretation of the encoding-retrieval match benefit, which would predict the opposite relationship, is not supported.

A key question of interest is whether increasing context variability benefits item memory because it increases the set of cues during encoding that will potentially be useful during retrieval. If the retrieval context is unknown or arbitrary, as in the Non-match condition, a wider variety of encoded cues might increase the likelihood that one of those cues will be reinstated during retrieval. If so, we might expect that high encoding variability would compensate for the lack of an explicit encoding-retrieval match. Indeed, the High Variability/Non-match condition (*M* = 0.86) produced approximately the same level of item memory as did the Low Variability/Match condition (*M* = 0.84, *t*(77) = 1.04, *p* = 0.30).

All differences in context variability were significant when compared within an encoding-retrieval match condition. The Same Context versus Low Variability comparisons were significant both when retrieval context matched (t(77) = 3.05, *p* < 0.005, d = 0.35) and when retrieval context did not match (t(77) = 5.13, *p* < 0.001, d = 0.58). The Low Variability versus High Variability comparisons were also significant for both matching encoding-retrieval contexts (t(77) = 2.49, *p* < 0.05, d = 0.28) and non-matching contexts (t(77) = 3.76, *p* < 0.001, d = 0.43). The overall effect of variability was smaller within the Match condition (High Variability/Match vs. Same Context/Match, *t*(77) = 5.62, *p* < 0.001, d = 0.64) than within the non-match condition (High Variability/Non-Match vs. Same Context/Non-Match, *t*(77) = 8.45, *p* < 0.001, d = 0.96). Thus, the benefit of context variability is greater when the encoding and retrieval contexts do not match but there is an additional benefit for variability even when the encoding and retrieval contexts do match. This may indicate that context variability has an additional mechanism to benefit item recognition beyond increasing the likelihood of an encoding-retrieval match.

In order to examine this potential additional mechanism, we conducted an exploratory analysis of the Low Variability/Match condition items based on the encoding features of the matching retrieval context that was presented. It should be noted that each comparison includes only 15 test trials per participant. The analysis indicated that there was a small numerical benefit for reinstatement of the twice-studied context (*M* = 0.89) over the once-studied context (*M* = 0.86) but that the difference was not statistically significant (*F*(1, 76) = 3.34, p = 0.07, ${\upeta }_{\text{p}}^{2}$ = 0.04). In addition, the overall effect of counterbalancing scheme (and therefore encoding order) on the Low Variability/Match items was not significant (*F*(1, 76) = 0.86, p = 0.36, ${\upeta }_{\text{p}}^{2}$ = 0.01). Finally, we compared whether reinstatement of the most recent encoding question for a Low Variability/Match item (recent reinstatement, *M* = 0.87) or an earlier encoding question (early reinstatement, *M* = 0.89) is more beneficial. The interaction between twice-studied or once-studied context reinstatement and A-A-B encoding order or A-B-B encoding order was not statistically significant (*F*(1, 76) = 0.92, p = 0.34, ${\upeta }_{\text{p}}^{2}$ = 0.01). We note that the current study was not specifically designed to test these differences and therefore these findings should be considered preliminary rather than conclusive.

### General discussion

The current study investigated whether encoding variability/context variability is a robust strategy for increasing recognition success. The four experiments reported here consistently demonstrated that increasing context variability during encoding, which was achieved by increasing the range of encoding questions used to study each item, improved recognition memory for the studied item. As reviewed in the *Introduction*, this finding is somewhat at odds with prior findings using free-recall paradigms.

For example, Zawadzka and colleagues (2021) found that increasing context variability during encoding did not improve free-recall performance although it did improve cued-recall performance when semantic cues were used. The authors concluded (Zawadzka et al., 2021, p. 1), “encoding variability promoted via different orienting tasks…fosters more elaborate encoding of semantic features. This augmented semantic component benefits memory performance only when a memory test is utilized that taps predominantly semantic features of memory representations, minimizing the role of contextual and relational factors.” They link this finding to Glenberg’s (1979) component-levels theory, which argues that memory tests of differing types can rely on distinct components of the memory representation. We agree that it is likely a difference between the requirements of free-recall tests and recognition tests that drives the differences in findings. Although our context variability manipulation is conceptually similar to those that have not produced a memory benefit for free-recall tasks, we found consistent benefits to item recognition using that manipulation.

We found that the benefits of context variability for recognition occurred even when repetition spacing was carefully controlled, in Experiments 2, 3, and 4, and when source memory judgments were not collected, in Experiments 3 and 4. This differs from the only prior study in the literature that manipulated context variability, controlled repetition spacing, and measured item recognition (Zhang & Hupbach, 2023), which found a benefit of context variability only when source judgments were collected. The authors proposed that repetition during encoding encourages participants to rely on familiarity, rather than recollection, during retrieval. They further suggested that if encoding variability’s benefit to recognition performance is driven by recollection, then that benefit would only be observed when participants are explicitly asked to search for recollective evidence during retrieval. However, our findings are not consistent with this conclusion because they indicate that benefits of context variability can be detected even when source judgments are not collected. Although we asked participants to report the study list for each remembered item in Experiments 1 and 2, we only asked for item recognition judgments in Experiments 3 and 4. Our results do not rule out the possibility that context variability primarily benefits recollection (and therefore context retrieval) because task characteristics other than retrieval instructions might have driven participants to rely on recollection to a greater degree in our study than in Zhang and Hupbach’s study.

Zhang and Hupbach (2023) proposed a second possible explanation for the differences in their findings across experiments: that ceiling effects may have played a role in their results. The results from Experiments 3 and 4 in the current study support that proposal. When we implemented a 24-h delay between encoding and retrieval to reduce overall recognition performance, we found substantially larger context variability benefits than when we used an immediate test procedure. Further support comes from the behavioral findings in a recent study from our lab (Lim et al., 2023), which found a robust item memory benefit for variable context encoding (Cohen’s d = 0.67) when participants were tested after a multi-day delay. Future studies should keep in mind that repeated encoding trials often push immediate recognition performance close to ceiling. Therefore, observing a difference in recognition due to context variability may only be possible when the task is made more difficult in some way. An alternative possible explanation for the increased effect size when a 24-h or more delay occurs between encoding and retrieval is that a factor introduced during the delay period increases the benefits of context variability: sleep, consolidation, interference, etc. We cannot differentiate between these possibilities with the current study.

#### The role of attention during encoding

We conclude from our data that encoding variability, as implemented via our context variability manipulation, benefits recognition because it increases the number and variety of potential retrieval cues for each item. However, an alternative mechanism for the effect is an overall reduction in attention across repetitions of an item when the same encoding question is asked repeatedly for that item. Although it is difficult to assess participants’ attention levels during memory encoding, there are some aspects of the current data set that provide insight into the role of attention during repeated trials. First, it is important to note that a minimum of 11 trials intervened between repetitions in the experiments reported here. The average repetition distance in Experiments 3 and 4 was 15 trials. We think this level of repetition spacing makes it unlikely that participants held the first encoding experience in working memory until the repetition occurred (therefore minimizing attention applied to the second exposure).

If working memory maintenance of prior items was not possible, we might expect that a repetition using an identical question could facilitate retrieval of the first encoding experience from long-term memory (i.e., “reminding”) and allow the participant to report the same response without further cognitive processing. In contrast, changing the phrasing of a question should require engagement of attention to formulate an answer even when an item has been processed previously with a similar question. In Experiment 1, encoding question sets 1 and 2 (see Table 1) used identical wording in the Low Variability condition but unique wording in the High Variability condition. Encoding question sets 3 and 4 used unique wording in both the High and Low Variability conditions. The overall difference between High Variability and Low Variability was numerically greater when averaged across question sets 1 and 2 (mean = 0.050) than across question sets 3 and 4 (mean = 0.032). This lends some support to the idea that identical encoding question wording reduces attention to the second and following repetitions. However, the current study was likely underpowered to detect any statistical differences due to question set. Future studies could intentionally investigate how changes in question wording or other parameters that modulate attention and reminding affect the context variability benefit for recognition.

#### Theoretical implications for a benefit of encoding context variability on recognition

The theoretical implications of an overall benefit for context variability on item memory tested via recognition can be examined via the lens of the encoding specificity principle. This idea argues that cognitive processing during an event determines what information will be included in the memory for that event (Tulving & Thomson, 1973). Varying the context by varying the encoding questions being answered produces more breadth of thought about each item across repetitions and therefore more breadth of information encoded in each representation. For example, the word “fish” studied with respect to its usefulness on a deserted island might provoke thoughts about fish being a nutritious food and about the uses of fish bones as tools. Repetition of that same item/question pairing might lead to additional thoughts (e.g., the need for a fishing rod to have a regular food source, lack of cooking resources meaning the fish would be eaten as sushi, etc.), but those thoughts would likely fall within the narrow range of ideas related to being stranded on a deserted island. In contrast, if the second and third presentations of the word “fish” were assigned to different contexts, the range of ideas considered would be broader (e.g., “Would it hurt if this item fell on your foot?”, many fish are not heavy, but they may be slimy or have sharp scales and fins; “Is this item all one color?”, goldfish are uniformly orange, but betta fish can be multicolored).

Whether the information included in the memory representations formed during encoding affects eventual retrieval success may depend on the retrieval environment. Experiments 1 and 2 did not specifically implement any of the encoding contexts during retrieval but variety among those contexts led to greater recognition success. In Experiments 3 and 4 we see that an arbitrary retrieval context (in the Non-match conditions in those experiments) produced the largest benefit for context variability. We propose that context variability is beneficial in a non-matching retrieval context because of the breadth of information encoded. Returning to the example above, if a participant is asked to recognize the word “fish” from the experiment within a non-matching context (“Have you been near this item recently?”), more breadth in the information encoded increases the likelihood that some aspect of that information will be relevant during retrieval. That is, thinking about one’s most recent physical interaction with fish might call to mind any of several trains of thought. One might think about a pet fish (potentially related to the thoughts that arose when judging the color of fish, goldfish vs. betta), a recent meal in which fish was the protein source (potentially related to the thoughts that arose about the usefulness of fish on a deserted island), or an encounter with a fish in a body of water (potentially related to the thoughts that arose when thinking about slimy or sharp scales and fins). In this example, breadth of thought across the encoding contexts increases the likelihood that the arbitrary retrieval context will reinstate some aspect of the encoded representation of “fish.” In comparison, the repeated encoding context of usefulness on a deserted island is related to only one of these arbitrary retrieval scenarios. Thus, when the retrieval context does not match an encoding context, greater breadth of information considered during encoding is likely to be beneficial. This is similar to prior studies of “elaboration” as an encoding strategy, in which the explicit goal is increasing the number of cues created during encoding (Benjamin & Bjork, 2000; Craik & Tulving, 1975).

However, when the retrieval context explicitly matches an encoding context, the cues presented during retrieval have the potential to reinstate similar thoughts to those that occurred during encoding regardless of context variability. Instead, what may differ between consistent context exposures and variable context exposures during encoding is the *strength* of the association between the information reinstated during retrieval and the encoding experience. Again, returning to the example above, asking whether “fish” is useful on a deserted island just prior to the recognition judgment is likely to produce thoughts that reinstate information from the encoding representation in both the Low Variability (where that question was asked during three study exposures) and High Variability conditions (where that question was asked during one study exposure). The train of thought: “sure, I could eat it and food will be scarce” might be part of three separate representations in the Low Variability condition or it might be a recurring (and therefore strengthened) component of a single representation containing all study experiences with that item. In comparison, that train of thought would be part of only one representation in the High Variability condition, with no strengthening beyond a single exposure. Nonetheless, our data (in both Experiments 3 and 4) do not indicate a “strength” advantage for Same Context encoding followed by a Match retrieval context as compared to Low Variability or High Variability encoding.

It is not clear, from an encoding specificity perspective, why additional context variability beyond an encoding context that matches the retrieval context would benefit item memory, as seen in Experiment 4. Our results indicated that repetitions in varying contexts beyond the eventual retrieval context (High Variability condition) rather than consistently within the eventual retrieval context (Same Context condition) produced higher recognition accuracy. This refutes a potential strict interpretation of encoding-retrieval match, which could be viewed as predicting that Same Context encoding is more beneficial than High Variability encoding when the repeated context will match the retrieval context. In addition, it suggests that a context variability benefit to recognition memory has multiple underlying mechanisms (not just increasing the breadth of cues and therefore the likelihood of encoding-retrieval match). One possibility is that the additional benefit of context variability beyond encoding-retrieval match is driven by attention during encoding, as discussed above.

In addition to implications for encoding-retrieval match, the current study may have implications for theories about how the nature of memory representations for repeated events might differ from those for novel events. Theorists have argued that increasing list length via new items has a bigger effect on memory than increasing list length via repetitions of previously presented items (Murnane & Shiffrin, 1991; Ratcliff et al., 1990). This suggests that repeated items do not produce traces with the same degree of independence or “newness” as new items. A wide variety of hypotheses have been proposed to explain how the memory representations or “traces” of repeated events differ from representations of two unrelated events.

Some theorists explicitly propose that repetitions of an event are accumulated in a single trace (Hintzman et al., 1973; Sahakyan & Malmberg, 2018) if attentional resources allow the prior experience to be accessed during the repetition, often termed “reminding” or “study-phase retrieval.” Hintzman (2010) conducted a series of experiments using judgments of recency that revealed independent recency judgments across item repetitions. Depending on repetition spacing, a repeated item might be judged as more recent or less recent, which Hintzman explained via a “recursive reminding” hypothesis of repetition. This hypothesis is also supported by earlier work demonstrating that judgments of spacing are more accurate for related words than unrelated words, suggesting that the second item calls to mind presentation of the earlier, highly related item and thus allows the participant to encode the relative familiarity or temporal judgment as part of the memory of the second item (Hintzman & Block, 1973). In the current study, reminding of the previous event may be more likely in the Same Context and Low Variability conditions than in the High Variability condition due to context reinstatement. If so, future studies might investigate whether judgments of relative temporal distance between repetitions are more accurate in those conditions.

Models like REM (Retrieving Effectively from Memory; Shiffrin & Steyvers, 1997) propose that the number of traces created by item repetition is determined by the contextual similarity across those repetitions. That is, an item repeated in a sufficiently similar circumstance will result in a single memory trace that includes both experiences but is more detailed and specific than a memory trace resulting from a single exposure, termed “differentiation.” However, differentiation of a single trace can be blocked (such that separate traces are encoded) by manipulating the context of the second presentation. A weakness of this proposal is that the circumstances under which a single trace will be differentiated versus multiple traces created are largely undefined. In the current study, REM might predict that separate traces are more likely to be created in the High Variability condition than in the Same Context condition. If so, then our data suggest that creation of multiple distinct memory traces is beneficial for recognition memory as compared to differentiation/strengthening of a single trace across repetitions.

Other theorists propose that repetitions of an event always produce separate traces. In this case, the memory representation of a first encoding experience is not changed when the event is repeated but that the memory representation of the second encoding experience includes any information about the first experience that is retrieved during encoding (e.g., Raaijmakers, 2003; Siegel & Kahana, 2014). The representation of the second presentation might then retain or emphasize the features that are common among the first and second experience. Competition trace theory (Yassa & Reagh, 2013) proposes that repeated encoding of an item creates competition between the representations of each event, with overlapping features across repetitions more likely to be preserved and features that change across repetitions likely to be lost. This results in generalization of the representations across contexts to some degree (sometimes termed “semanticization”, e.g. Nelson & Shiffrin, 2013). Alternatively, the representation of the second presentation could retain or emphasize the features that are distinct in each experience. The current study did not investigate context memory performance and therefore cannot indicate whether context knowledge is more likely to be lost after variable encoding; however, recent work from other labs has investigated this proposal (e.g. Zhang & Hupbach, 2023).

## Conclusions

We propose that the current study refutes prior claims that encoding variability has null or negative effects on episodic memory. Instead, we found that encoding/context variability specifically benefits item recognition (in addition to prior research suggesting that variability can benefit cued recall when semantic cues are used). We found the item memory benefit of manipulating encoding question variability is consistent across a range of moderate to long repetition spacing conditions. We proposed that increasing encoding question variability might increase the number and variety of cues associated with an item, thereby increasing the likelihood of a match between encoding context and an unpredictable retrieval context. We found partial support for that mechanism (in that enforcing an encoding-retrieval context match reduced the overall benefit of increasing encoding variability). However, we also found that increasing variability benefited item memory even when that variability did not explicitly increase the match between encoding and retrieval circumstances. Therefore, we propose that additional mechanisms by which encoding variability across repetitions benefits item memory are yet to be identified.

## Data Availability

The data and materials for all experiments are available at 10.17605/OSF.IO/JU2WX.

## Footnotes

1 Other studies compare massed (back-to-back, without any intervening items) encoding exposures with spaced encoding exposures (in which other items are encoded between the exposures). Items encoded across massed trials may suffer from waning attention. However, the benefit of increasing the number of intervening items from short (but not massed) spacing to long spacing is not so obvious. These are the circumstances under which encoding variability or temporal context variability have been proposed as a mechanism for the spacing effect (e.g., Bower, 1972; Melton, 1970).

2 No item-recognition judgments were collected in Experiment 3 and therefore we will not discuss it further.

## Code availability

Custom Matlab scripts for all experiments are available at 10.17605/OSF.IO/JU2WX.

## Declarations

### Open practices statement

The data and materials for all experiments are available at 10.17605/OSF.IO/JU2WX. Experiment 4 was preregistered at 10.17605/OSF.IO/SU4DB.

## References

[CR1] Anderson, J., & Reder, L. (1979). An Elaborative Processing Explanation of Depth Processing. *L.S. Cermak & F.I.M. Craik. (Eds.), Levels of Processing in Human Memory*.

[CR2] Benjamin, A. S., & Bjork, R. A. (2000). On the relationship between recognition speed and accuracy for words rehearsed via rote versus elaborative rehearsal. *Journal of Experimental Psychology: Learning, Memory, and Cognition,**26*(3), 638–648. 10.1037/0278-7393.26.3.638 10855422 10.1037//0278-7393.26.3.638

[CR3] Benjamin, A. S., & Tullis, J. (2010). What makes distributed practice effective? *Cognitive Psychology,**61*(3), 228–247. 10.1016/j.cogpsych.2010.05.004 20580350 10.1016/j.cogpsych.2010.05.004 PMC2930147

[CR4] Bjork, R. A., & Bjork, E. L. (1992). A new theory of disuse and an old theory of stimulus fluctuation. In *Essays in honor of William K. Estes, Vol. 1: From learning theory to connectionist theory; Vol. 2: From learning processes to cognitive processes* (pp. 35–67). Lawrence Erlbaum Associates, Inc.

[CR5] Bower, G. H. (1972). Stimulus-sampling theory of encoding variability. In A. W. Melton & E. Martin (Eds.), *Coding processes in human memory* (Vol. 3, pp. 85–123). Winston.

[CR6] Bray, J. F., Robbins, D., & Witcher, W. B. (1976). Encoding variability theory and the spacing effect in associate learning. *Memory & Cognition,**4*(5), 548–552. 10.3758/BF03213217 21286980 10.3758/BF03213217

[CR7] Brysbaert, M., Mandera, P., McCormick, S. F., & Keuleers, E. (2019). Word prevalence norms for 62,000 English lemmas. *Behavior Research Methods,**51*(2), 467–479. 10.3758/s13428-018-1077-9 29967979 10.3758/s13428-018-1077-9

[CR8] Brysbaert, M., & New, B. (2009). Moving beyond Kučera and Francis: A critical evaluation of current word frequency norms and the introduction of a new and improved word frequency measure for American English. *Behavior Research Methods,**41*(4), 977–990. 10.3758/BRM.41.4.977 19897807 10.3758/BRM.41.4.977

[CR9] Brysbaert, M., Warriner, A. B., & Kuperman, V. (2014). Concreteness ratings for 40 thousand generally known English word lemmas. *Behavior Research Methods,**46*(3), 904–911. 10.3758/s13428-013-0403-5 24142837 10.3758/s13428-013-0403-5

[CR10] Cepeda, N. J., Pashler, H., Vul, E., Wixted, J. T., & Rohrer, D. (2006). Distributed practice in verbal recall tasks: A review and quantitative synthesis. *Psychological Bulletin,**132*(3), 354–380. 10.1037/0033-2909.132.3.354 16719566 10.1037/0033-2909.132.3.354

[CR11] Coltheart, M. (1981). The MRC psycholinguistic database. *Quarterly Journal of Experimental Psychology,**33A*, 497–505.

[CR12] Cooper, E., Greve, A., & Henson, R. N. (2019). Little evidence for Fast Mapping (FM) in adults: A review and discussion. *Cognitive Neuroscience,**10*(4), 196–209. 10.1080/17588928.2018.1542376 30451079 10.1080/17588928.2018.1542376 PMC6711760

[CR13] Cousineau, D. (2005). Confidence intervals in within-subject designs: A simpler solution to Loftus and Masson’s method. *Tutorials in Quantitative Methods for Psychology*, *1*(1), 42–45. 10.20982/tqmp.01.1.p042

[CR14] Coutanche, M. N., & Thompson-Schill, S. L. (2014). Fast Mapping Rapidly Integrates Information into Existing Memory Networks. *Journal of Experimental Psychology. General,**143*(6), 2296–2303. 10.1037/xge0000020 25222265 10.1037/xge0000020 PMC4244253

[CR15] Craik, F. I. M., & Tulving, E. (1975). Depth of processing and the retention of words in episodic memory. *Journal of Experimental Psychology: General,**104*(3), 268–294. 10.1037/0096-3445.104.3.268

[CR16] Delaney, P. F., Verkoeijen, P. P. J. L., & Spirgel, A. (2010). Chapter 3 - Spacing and Testing Effects: A Deeply Critical, Lengthy, and At Times Discursive Review of the Literature. In B. H. Ross (Ed.), *Psychology of Learning and Motivation* (Vol. 53, pp. 63–147). Academic Press. 10.1016/S0079-7421(10)53003-2

[CR17] Dennis, S., & Humphreys, M. S. (2001). A context noise model of episodic word recognition. *Psychological Review,**108*(2), 452–478. 10.1037/0033-295X.108.2.452 11381837 10.1037/0033-295x.108.2.452

[CR18] Estes, W. K., & Burke, C. J. (1953). A theory of stimulus variability in learning. *Psychological Review,**60*(4), 276–286. 10.1037/h0055775 13089006 10.1037/h0055775

[CR19] Glenberg, A. M. (1979). Component-levels theory of the effects of spacing of repetitions on recall and recognition. *Memory & Cognition,**7*(2), 95–112. 10.3758/BF03197590 459836 10.3758/bf03197590

[CR20] Greenberg, D. L., & Verfaellie, M. (2010). Interdependence of episodic and semantic memory: Evidence from neuropsychology. *Journal of the International Neuropsychological Society : JINS,**16*(5), 748–753. 10.1017/S1355617710000676 20561378 10.1017/S1355617710000676 PMC2952732

[CR21] Greene, R. L., & Stillwell, A. M. (1995). Effects of encoding variability and spacing on frequency discrimination. *Journal of Memory and Language,**34*(4), 468–476. 10.1006/jmla.1995.1021

[CR22] Hintzman, D. L. (2010). How does repetition affect memory? Evidence from judgments of recency. *Memory & Cognition,**38*(1), 102–115. 10.3758/MC.38.1.102 19966243 10.3758/MC.38.1.102

[CR23] Hintzman, D. L., & Block, R. A. (1973). Memory for the spacing of repetitions. *Journal of Experimental Psychology,**99*(1), 70–74. 10.1037/h0034761

[CR24] Hintzman, D. L., Block, R. A., & Summers, J. J. (1973). Modality tags and memory for repetitions: Locus of the spacing effect. *Journal of Verbal Learning and Verbal Behavior,**12*(2), 229–238. 10.1016/S0022-5371(73)80013-1

[CR25] Huff, M. J., & Bodner, G. E. (2014). All varieties of encoding variability are not created equal: Separating variable processing from variable tasks. *Journal of Memory and Language,**73*, 43–58. 10.1016/j.jml.2014.02.004 25018583 10.1016/j.jml.2014.02.004 PMC4088266

[CR26] Jacoby, L. L., & Craik, F. I. M. (1979). Effects of Elaboration of Processing at Encoding and Retrieval: Trace Distinctiveness and Recovery of Initial Context. In *Levels of Processing in Human Memory (PLE: Memory)*. Psychology Press.

[CR27] Karpicke, J. D., Lehman, M., & Aue, W. R. (2014). Retrieval-based learning: An episodic context account. In *The psychology of learning and motivation, Vol. 61* (pp. 237–284). Elsevier Academic Press.

[CR28] Kucera, H., & Francis, W. N. (1967). *Computational analysis of present-day American English*. Brown University Press.

[CR29] Lim, Y.-L., Lang, D. J., & Diana, R. A. (2023). Cognitive tasks affect the relationship between representational pattern similarity and subsequent item memory in the hippocampus. *NeuroImage,**277*, 120241. 10.1016/j.neuroimage.2023.120241 37348623 10.1016/j.neuroimage.2023.120241

[CR30] Lohnas, L. J., Polyn, S. M., & Kahana, M. J. (2011). Contextual Variability in Free Recall. *Journal of Memory and Language,**64*(3), 249–255. 10.1016/j.jml.2010.11.003 21379369 10.1016/j.jml.2010.11.003 PMC3046415

[CR31] McClelland, J. L., McNaughton, B. L., & O’Reilly, R. C. (1995). Why there are complementary learning systems in the hippocampus and neocortex: Insights from the successes and failures of connectionist models of learning and memory. *Psychological Review,**102*(3), 419–457. 7624455 10.1037/0033-295X.102.3.419

[CR32] Melton, A. W. (1970). The situation with respect to the spacing of repetitions and memory. *Journal of Verbal Learning and Verbal Behavior,**9*(5), 596–606. 10.1016/S0022-5371(70)80107-4

[CR33] Morey, R. D. (2008). Confidence Intervals from Normalized Data: A correction to Cousineau (2005). *Tutorials in Quantitative Methods for Psychology*, *4*(2), 61–64. 10.20982/tqmp.04.2.p061

[CR34] Morris, C. D., Bransford, J. D., & Franks, J. J. (1977). Levels of processing versus transfer appropriate processing. *Journal of Verbal Learning and Verbal Behavior,**16*(5), 519–533. 10.1016/S0022-5371(77)80016-9

[CR35] Murnane, K., & Shiffrin, R. M. (1991). Interference and the representation of events in memory. *Journal of Experimental Psychology: Learning, Memory, and Cognition,**17*(5), 855–874. 10.1037/0278-7393.17.5.855 1834768 10.1037//0278-7393.17.5.855

[CR36] Nelson, A. B., & Shiffrin, R. M. (2013). The co-evolution of knowledge and event memory. *Psychological Review,**120*(2), 356–394. 10.1037/a0032020 23458086 10.1037/a0032020

[CR37] Opitz, B. (2010). Context-dependent repetition effects on recognition memory. *Brain and Cognition,**73*(2), 110–118. 10.1016/j.bandc.2010.04.003 20493623 10.1016/j.bandc.2010.04.003

[CR38] Postman, L., & Knecht, K. (1983). Encoding Variability and Retention. *Journal of Verbal Learning and Verbal Behavior,**22*(2), 133–152.

[CR39] Raaijmakers, J. G. (2003). Spacing and repetition effects in human memory: Application of the SAM model. *Cognitive Science,**27*(3), 431–452.

[CR40] Ratcliff, R., Clark, S. E., & Shiffrin, R. M. (1990). List-strength effect: I. Data and discussion. *Journal of Experimental Psychology: Learning, Memory, and Cognition*, *16*(2), 163–178. 10.1037/0278-7393.16.2.163 2137859

[CR41] Ross, B. H., & Landauer, T. K. (1978). Memory for at least one of two items: Test and failure of several theories of spacing effects. *Journal of Verbal Learning & Verbal Behavior,**17*(6), 669–680. 10.1016/S0022-5371(78)90403-6

[CR42] Sahakyan, L., & Malmberg, K. J. (2018). Divided attention during encoding causes separate memory traces to be encoded for repeated events. *Journal of Memory and Language,**101*, 153–161. 10.1016/j.jml.2018.04.004

[CR43] Shiffrin, R. M., & Steyvers, M. (1997). A model for recognition memory: REM - retrieving effectively from memory. *Psychonomic Bulletin & Review,**4*, 145–166. 21331823 10.3758/BF03209391

[CR44] Siegel, L. L., & Kahana, M. J. (2014). A retrieved context account of spacing and repetition effects in free recall. *Journal of Experimental Psychology: Learning, Memory, and Cognition,**40*, 755–764. 10.1037/a0035585 24564545 10.1037/a0035585 PMC4288756

[CR45] Sievers, C., Bird, C. M., & Renoult, L. (2019). Predicting memory formation over multiple study episodes. *Learning & Memory (Cold Spring Harbor, N.Y.)*, *26*(12), 465–472. 10.1101/lm.049791.119 10.1101/lm.049791.119 PMC6859827 31732707

[CR46] Smith, S. M., & Handy, J. D. (2016). The crutch of context-dependency: Effects of contextual support and constancy on acquisition and retention. *Memory,**24*(8), 1134–1141. 10.1080/09658211.2015.1071852 26247606 10.1080/09658211.2015.1071852

[CR47] Tulving, E., & Thomson, D. M. (1973). Encoding specificity and retrieval processes in episodic memory. *Psychological Review,**80*, 352–373.

[CR48] Verkoeijen, P. P. J. L., Rikers, R. M. J. P., & Schmidt, H. G. (2004). Detrimental Influence of Contextual Change on Spacing Effects in Free Recall. *Journal of Experimental Psychology: Learning, Memory, and Cognition,**30*(4), 796–800. 10.1037/0278-7393.30.4.796 15238024 10.1037/0278-7393.30.4.796

[CR49] Watkins, O. C., & Watkins, M. J. (1975). Buildup of proactive inhibition as a cue-overload effect. *Journal of Experimental Psychology: Human Learning and Memory,**1*(4), 442–452. 10.1037/0278-7393.1.4.442

[CR50] Yassa, M. A., & Reagh, Z. M. (2013). Competitive trace theory: A role for the hippocampus in contextual interference during retrieval. *Frontiers in Behavioral Neuroscience*, *7*. 10.3389/fnbeh.2013.00107 10.3389/fnbeh.2013.00107 PMC3740479 23964216

[CR51] Zawadzka, K., Baloro, S., Wells, J., Wilding, E. L., & Hanczakowski, M. (2021). On the memory benefits of repeated study with variable tasks. *Journal of Experimental Psychology: Learning, Memory, and Cognition*, 1067–1082. 10.1037/xlm0001013 10.1037/xlm0001013 33591775

[CR52] Zhang, M., & Hupbach, A. (2023). The effects of variable encoding contexts on item and source recognition. *Memory & Cognition,**51*(2), 391–403. 10.3758/s13421-022-01353-8 35980546 10.3758/s13421-022-01353-8

